# Supplementary material for: Integrating postpartum IUD counselling and insertion into routine maternity care in Nepal: Assessing trends over time
Source: PLOS Glob Public Health. 2023 Mar 22;3(3):e0001665. doi: 10.1371/journal.pgph.0001665 (PMC10032507; doi:10.1371/journal.pgph.0001665)
Supplement: Attachment — Submitted filename: Response to reveiwers.docx [file pgph.0001665.s003.docx]

**Points by points responses to editors and reviewers**

Journal Requirements:

1. Please provide a detailed online Financial Disclosure statement. This is published with the article. It must therefore be completed in full sentences and contain the exact wording you wish to be published.

a. Please clarify all sources of funding (financial or material support) for your study. List the grants (with grant number) or organizations (with url) that supported your study, including funding received from your institution. 
b. State the initials, alongside each funding source, of each author to receive each grant.
c. State what role the funders took in the study. If the funders had no role in your study, please state: “The funders had no role in study design, data collection and analysis, decision to publish, or preparation of the manuscript.”
d. If any authors received a salary from any of your funders, please state which authors and which funders.

If you did not receive any funding for this study, please simply state: “The authors received no specific funding for this work.”

**Response: Online Financial disclosure statement has been provided on our original submission.**

2. Please update your online Competing Interests statement. If you have no competing interests to declare, please state: “The authors have declared that no competing interests exist.”

**Response: We have updated Competing interests statement.**

3. Please provide separate figure file in .tif or .eps format and ensure that all files are under our size limit of 10MB.

For more information about how to convert your figure files please see our guidelines: <https://journals.plos.org/globalpublichealth/s/figures>

**Response: Separate .tif format figure are submitted.**

4. Please ensure that you refer to Figures 1 and 3 in your text as, if accepted, production will need these references to link the reader to the figures.

**Response: We doubled checked about this.**

5. We have noticed that you have uploaded Supporting Information files, but you have not included a list of legends. Please add a full list of legends for your Supporting Information files after the references list.

**Response: Legend for your supplementary is provided**

Additional Editor Comments (if provided):

Reviewer #1: Integrating PPIUD counselling and insertion into routine maternity care in nepal: are effects sustainable?

Overall
Thank you for your manuscript. The data is valuable and some of the interpretations are interesting but there is some clear information missing which needs to be addressed before conclusions can be made. Please see my detailed comments.

As I understand it there are two sets of analysis - one is looking at comparing the two groups and the second is looking at changes over time. As I right in thinking there was no change in the intervention and things were being implemented in the same way in 2017 as they were in July 2018? If this is the case, then the discussions about sustainability are not really valid, as you cant discuss sustainability if the support was not removed. As the latter would be looking at what happens once all interventions are removed. Maybe it would be better to centre the discussion around a longitudinal analysis of the intervention over time and remove the word and discussion about sustainability.

**Response: Thank you for the important comment. We understand that almost all intervention activities were withdrawn at the end of the study period. However, one counsellor (reduced from two during the intervention) continued to be employed in each participating hospital. We have revised the text and do not interpret results in terms of sustained impact. We also revised the title of the manuscript as “Integrating postpartum IUD counselling and insertion into routine maternity care in Nepal: Assessing trends over time” to reflect this revision.**

Please highlight as a limitation the fact that you looked at counselling and insertion and not counselling and acceptance/consent. The latter is a more direct result of counselling where as insertion has a further rate-limiting factor which you are not accounting for, which is the availability of a trained provider to insert at the time of delivery. In other words amongst this data you cannot ascertain the number of women consenting who did not end up receiving PPIUD even though they may have wanted one.

**Response: Thank you for highlighting this. It has now been added to the limitations.**

Abstract
Please amend the abstract according to the suggested changes in the detailed review of the manuscript as I don't believe some of your conclusions hold.
Eg: 'The results suggest that the impact and sustained effects of the intervention could be enhanced by regular follow up and supervision, expanding the coverage of PPFP counselling to include other health facilities, and improving the training for providers, especially on providing high quality PPFP counselling.'
The results don't suggest that. You have no data to suggest that the sustainability would be improved by regular follow up and supervision etc… This is your opinion or recommendation. You need to be clear about what your results show and what you suggest/advise.

**Response: We agree and have revised the text. For example, the above conclusion has been revised as**

**“The results suggest that counselling was well integrated with the maternity care, though uptake of PPIUD dropped after intervention activities such as active monitoring, technical supervision, provision of IUDs and training were withdrawn”.**

Background
Line 105 - What is the purpose of comparing the two groups? Why does it matter. Please expand. What are the differences between the two groups? Are there any structural differences? It seems to me that the main difference is the implementation period. Please highlight this.

**Response: Thank you for raising this point. Indeed, the comparison is between the baseline and intervention periods and not between two (control and intervention) groups. We have revised the text as follows:**

***“This study also aims to examine the longer-term intervention effects using follow-up data for the month of July 2018 (15 months after the end of the evaluation study enrolment when almost all of the intervention activities were no longer operational). This follow-up data will give an indication of whether PPIUD counselling, PPIUD insertion and insertion among those counselled has increased or decreased since the original analysis. It is important to investigate the longer-term impact of the intervention to examine if the outcomes observed at the end of the intensive intervention were maintained 15 months later when most of its active elements were no longer operational. Such understanding will assist with the design of programs and strategies to integrate PPFP and PPIUD counselling and PPIUD insertion into existing antenatal and institutional delivery services in Nepal*”.**

**Please see line 110-119.**

Line 106 - What do you mean by '15 months after the end of the evaluation study enrolment'. What is the significance of this time period. Was that the end of the intervention period? What interventions ended at this point? Did funding end? Did training of staff end at this point? What support was withdrawn? Please explain in more detail. It seems to me that later on you mention the intervention is still ongoing until June 2019.

**Response: The study enrolment and nearly all of the intervention activities stopped at the end of the enrolment period in February 2017. However, one of the two counsellors working during the intervention period was retained and working after the end of the intervention. We have revised the relevant text. See line 110-113.**

Data and Methods
Line 116
Please reference FIGO's PPIUD initiative rather than just explaining it in your own words without a reference. This paper should help: 'Planning and implementation of a FIGO postpartum intrauterine device initiative in six countries' Linda de Caestecker et al in the IJGO

**Response: Thank you for providing the reference. We have reviewed and cited it. See line 124-145.**

Line 132 The evaluation applied a cluster-randomized stepped-wedge trial design.
Why did you choose this design? Why were the hospitals grouped as such. What was the significance between the two groups. Please give more information on the details of each of the hospitals in the two groups. Were they comparable in terms of structure? Human Resources / number births / demographics of women using the hospitals / facilities for counselling and insertion etc…

**Response: Thank you for raising this important question. We randomized at the cluster level to avoid the spillover effects of the intervention. We used a stepped-wedge trial design considering the potential benefits of the intervention to all women. Thus, a cluster-randomized stepped-wedge trial design is the most appropriate.**

**Six local hospitals were selected as clusters that were: (1) tertiary health facilities with large catchment areas; (2) having 6,000 or more obstetric caseloads per year; and (3) not yet providing PPIUD services. Six hospitals were grouped as three pairs based on their annual number of deliveries. Within each pair, one hospital was randomized into Group 1, and the other into Group 2. The two hospitals in each pair were broadly similar in terms of client demographics, annual number of births, structure and human resources at the baseline.**

**We added the information above to the manuscript. See line 157-165.**

Line 139 You mention the intervention ended in June 2019. What was the purpose of analysing the effect after 'the enrolment period'. What changed after the end of this period? Did the interventions change in some way? If not, then I suggest your analysis is more of an interpretation over time, looking at the longitudinal impact of the intervention over all the months.

**Response: Thank you for raising this point. The purpose of analyzing the effect after “the enrolment period” is to test the longer term impact of the FIGO intervention. After the end of this period, FIGO intervention component of training, technical supervision and technical support were stopped in Groups 1 and Group 2 hospitals. However, the information materials were left on site and one counsellor continued to serve in each of the six participating hospitals. We have clarified that in the manuscript as follows:**

**“*The evaluation study had an 18-month enrollment period, from September 2015 to February 2017. Group 1 hospitals and Group 2 hospitals initiated the FIGO intervention three months and nine months, respectively, after the beginning of the enrollment data collection and maintained the intervention status since then. After the 18-month enrollment period, the evaluation study stopped surveying new patients on a regular basis while FIGO continued to receive data on the number of deliveries, and number of women counselled and had PPIUD inserted*. *FIGO intervention activities including training of providers, monitoring and technical supervision were stopped and the number of counsellors was reduced from two to one per hospital”***.

**We also clarified that our analysis is an interpretation over time by adding the following sentence under the statistical methods part: “*We analyzed the longitudinal impact of the intervention by focusing on four crucial months – the first month of the baseline (September 2015), the middle month of the intervention (July 2016 for Group 1 hospitals and October 2016 for Group 2 hospitals), the end month of the evaluation study enrollment (February 2017), and the additional one month in July 2018.”***

Line 124
I don't believe the FIGO PPIUD initiative supplied Cu IUDs - these were supplied by the government only and not through the initiative. Please use information re implementation from the implementers descriptive paper. This will ensure it is accurate.

**Response: Thank you for the suggestion. PPIUD was indeed provided by the Government with FIGO/NESOG following up with the Government to ensure regular supply. We have taken into account the information provided in the descriptive paper on the implementation by implementers.**

Statistical methods

Line 190 - 193: Please change this paragraph and describe it as a longitudinal assessment at 4 points. The way it is currently described makes it look like the final assessment is at the end of the intervention, which I think is not the case?

**Response: Thank you. We deleted this paragraph and revised the following sentence by highlighting it is a longitudinal assessment at 4 points.**

***“We analyzed the longitudinal impact of the intervention by focusing on four crucial points – the first month of the baseline (September 2015), the middle month of the intervention (July 2016 for Group 1 hospitals and October 2016 for Group 2 hospitals), the end month of the evaluation study enrollment (February 2017), and the additional one month in July 2018.*”**

Results
Line 212: Regarding demographics, I suggest you run a statistical analysis comparing your analysis groups to your baseline group as otherwise it is not certain that the groups are similar enough for comparison. Parity as you say is grossly dissimilar in July 2018. Why did this happen? You have interviewed a cohort of women with very different contraceptive intentions than you have at baseline. It makes comparing them impossible. One possibility would be to separate out from your other groups the Para 1s and seeing if your differences hold? If you cant do this it may be better to leave this months data out completely.

**Response: Thank you! We have conducted Chi-square tests for Table 1 comparing baseline with each of the later three months. Overall, socio-demographics across months are similar. Please refer to Revised Table 1.**

Line 272 - I think you mean figure 3 not 2.

**Response: Many thanks for spotting this. It has been changed from Figure 2 to Figure 3.**

Tables 2 and 3
Unless there is something specific that happened in 02/2017 I’m not sure of the significance of comparing that point to one month in July 2018? Stats looking at longitudinal changes may be more valuable?

**Response: Thank you for raising an important point. We have used 02/2017 because it was the last month for the data collection of the study. For the main evaluation study, 02/2017 was the end date when all of the data collection and most of the intervention activities stopped. It is, for this reason, we compare changes between 02/2017 and July 2018. We have point estimates in Tables and longitudinal monthly trends in Figures. Note that no monthly data were available between 02/2017 and July 2018 as no such data were collected.**

Discussion
Line 345 - You may be interested in three further studies looking exactly at the issue you mention. The more recent studies involved one of your group 1 hospitals (Koshi) and reported on the involvement of Female Community Health Volunteers, which improved uptake and outcomes in counselling and uptake in the surrounding hospitals. Please read these studies as they are relevant to your points about ANC counselling vs in hospital counselling.

K Thapa, R Dhital, S Rajbhandari, S Acharya, S Mishra, S Mani Pokhrel, S Pande, E-A Tunnacliffe, A Makins. Factors affecting the behavior outcomes on post-partum intrauterine contraceptive device uptake and continuation in Nepal: a qualitative study. BMC Pregnancy and Childbirth 2019; 19: 148
Rolina Dhital, Ram Chandra Siwal, Khem Narayan Pokhrel, Sabina Pokhrel, Heera Tuladhar, Suzanna Bright, Emily-Anne Tunnacliffe, Kusum Thapa, Anita Makins. Evaluating the impact of female community health volunteer involvement in a postpartum family planning intervention in Nepal: a mixed-methods study at one-year post-intervention. PLOS ONE. Published October 20, 2021. Available: [https://doi.org/10.1371/journal.pone.0258834](https://urldefense.proofpoint.com/v2/url?u=https-3A__doi.org_10.1371_journal.pone.0258834&d=DwMF-g&c=WO-RGvefibhHBZq3fL85hQ&r=j8I2Pn943OHozli27HANVuFGAA3lMF9nFhqWgwBXGHU&m=5kyr49FYr4j1BJvm36zc72xeiEobdAp87f8OhKCqW3hOmuKODidqK_o7RoGV_dM3&s=Gg_F9RF0PdtU_LUfJFElKNkGlF0clhYXdWE-Ty9TfDs&e=)
K. Thapa, R. Dhital, Sameena Rajbhandari, Sangeeta Mishra, Shanti Subedi, Bhogendra Dotel, Sapana Vaidya, Saroja Pande, Emily-Anne Tunnacliffe, Anita Makins and Sabaratnam Arulkumaran. Improving post-partum family planning services provided by female community health volunteers in Nepal: a mixed methods study. BMC Pregnancy and Childbirth 2020; 20:123

**Response: We are grateful for your kindly providing useful references. They have been incorporated into different parts of the discussion section.**

Line 368 - I think you mean PPIUD insertion among women in study hospitals dropped as there is no information on consent. Consenting and receiving PPIUD are two different things. My understanding is that you measured insertion. Please correct this.

**Response: We agree with the comment. The word ‘acceptance’ has now been replaced by ‘insertion’ or ‘uptake’ throughout the manuscript.**

Line 368 - Your one month follow up group is entirely different to your other groups as 99% are Para 1s - i.e. only just had one baby. In low income countries the outcome of that birth is more uncertain and much fewer women who have only just given birth to their first child are likely to accept contraception. You are not comparing similar groups of women.

**Response: We agree. We have rechecked the analysis of data and found that percent distribution by parity and ethnicity in July 2018 shown in the previous version were in error. We have corrected the error and revised Table 1. Also, please see the response to the comment below.**

Paragraph 368 to 384 - I don't think you can suggest a true difference between PPIUD insertion rates in the group based on two major flaws:
1. The fact that there is such a high level of fluctuation from month to month and you only have a snap shot of one month.
2. The fact that the groups are different. Your 2018 group is 99% Para 1s. These women are not comparable to your original group.
You mention these as limitations but in actual fact in makes it impossible to draw any firm conclusions. As mentioned earlier you could try and make a comparison between Para 1s in the other groups in order to get round issue 2. Issue 1 remains. IT be worth just removing that data?

**Response: Thank you for the thoughtful comment. We have redone the analysis of data and found that percent distribution by parity and ethnicity in July 2018 shown in the previous version were in error. The corrected Table 1 is now included. Parity distribution in July 2018 is broadly comparable. Therefore, the July 2018 results can be compared with other periods. We sincerely apologize for the error in Table 1 that was originally included in the manuscript.**

Line 396
The data doesn't suggest this. You are suggesting that as a possible explanation - that the group 2 hospitals may have had these issues. Please word this correctly. And please expand and substantiate your suggestion with information regarding the differences in the structures of the hospitals in the two groups. What was the staff to patient ratio? Which hospitals had individual private counselling spaces? Which hospitals had lack of IEC material or shortages of IUDs? What were their management structures? If you don't have this information please explain this as a limitation of your work and hence ability to interpret the results.

**Response: Thank you for raising this important point. The study hospitals within the pair were broadly comparable at baseline. However, Group 2 hospital had a shorter exposure period to full intervention than Group 1 which may have affected strengthening system readiness, including the provider’s capacity to deliver care and quality counselling and insertion services.  We have revised the manuscript to clarify this point.**

Line 412 to 415
I would go as far as to say that the data from July 2018 is not assessing sustainability as my understanding is that intervention was still on going. The question is why its impact was not as great as first off.

**Response: The intervention was curtailed to one counsellor while all other components including training of providers were dropped. We have amended the text and no longer speak of sustainability or sustained impact of the intervention**.

Line 418
What is the purpose of comparing the two groups? My understanding is that it was a cluster randomized study as so the two groups would not be different. If this is the case what is the value in comparing the two groups? Is it the length of time of implementation from your analysis? If so, please say this.

**Response: We compare two groups because of the difference in period of exposure to intervention. As the findings show, Group 2 with shorter period of exposure made slower progress than Group 1 hospitals. Also, the mid-month of intervention varied between the two group and, therefore, it was necessary to keep the two separate.**

Line 420
The study as it stands is not sufficient to look at sustainability. Please amend.

**Response: Thank you. We agree and have amended the text accordingly.**

Line 424/5
'…impact was notable and seemingly deteriorated during the follow up'. You are not able to conclude this with the methodology as it stands.

**Response: Noted with thanks and amended the text.**

Line 429

There is little information in your study about the training of providers and how it was lacking. I suggest you expand on this in the methodology section. As its difficult to suggest changes when you haven't included any information on what the training involved and how it could be of a higher standard. Perhaps you are referring to results from another study? If so please explain this.

**Response: Instead of recommending that training be improved, we are recommending that refresher training is provided – as considered in the context of Nepal and together with findings from other studies in the country.**

Conclusion
Line 440 - 445 Same issue as I have raised above.

Line 445 - From the information provided in the paper, this is the only plausible explanation for the differences in the 2 groups and should be highlighted as the main conclusion of that analysis. The remainder you mention is your opinion, and needs to be explained as such. Unless you are able to substantiate the other claims with more data / evidence.

**Response: Recommendations in the conclusion have been revised.**

Figures
Fig 3 and 4 - the variations from one month to the next are enormous in both groups. There must be some implementation reason for this as the counselling rates are much more smooth - eg were their sufficient trained inserters at all times day and night (that may explain the drops) / were their stock outs of IUDs (would also explain the drops), were insertions immediately postpartum(ie in the labour room) or within 48hrs meaning availability of an area on the ward to do it may have limited insertion? Please explain further. If you do not have this information please explain as a limitation when interpreting these two graphs.

**Response: Thank you. We have revised the text and added the following information among the limitations of this study.**

**“*Another limitation is that this study looks at counselling and insertion and not counselling and acceptance or consent to the PPIUD insertion. This limits the analysis to those who had the PPIUD inserted and does not account for those who had consented to having a PPIUD but were unable to procure it, due to the unavailability of a trained provider and/or the PPIUD at the time of delivery, for example. The latter would be a better indicator to monitor PPIUD counselling impact in terms of generating the demand for PPIUD.***

***With regards to assessing the longer term impact of the intervention, the sample used data from July 2018 have given a glimpse for a selected month 15 months after the end of study enrolment and a rigorous implementation of the intervention. However, a longer observational period is needed to determine the sustained impact of the intervention beyond its implementation period. Lastly, due to the lack of data, we were unable to consider information critical to explaining the levels and trends over time and by hospital group. More specifically, no information was available on any stock out of supplies, including PPIUD, availability of trained staff for IUD insertion at the time of delivery, transfer of trained staff from study to non-study hospitals and on other potential confounders."***

**Please see lines 481-497.**
