# Supplementary material for: Integrating postpartum IUD counselling and insertion into routine maternity care in Nepal: Assessing trends over time
Source: PLOS Glob Public Health. 2023 Mar 22;3(3):e0001665. doi: 10.1371/journal.pgph.0001665 (PMC10032507; doi:10.1371/journal.pgph.0001665)
Supplement: Attachment — Submitted filename: Response to Reviewers.docx [file pgph.0001665.s004.docx]

**Points by points responses to editors and reviewers**

Reviewer #2: This is an important topic both in that uptake of PPIUD is needed, and also that it is important for us to look at if, and also what can make, our interventions sustainable without program support. This article increases the knowledge in some areas. Notable to me is that if teaching maintains and uptake decreases something must have changed in the teaching...

Response: Thank you.

**Reviewer #3:** This manuscript describes an analysis looking at long-term impact of an intervention to promote PPIUD in Nepal. The manuscript has been edited in response to reviewer comments once; I have some additional suggestions below:

Abstract

See point below.

**Introduction**

It would seem that the framing of this manuscript could relate more directly to the aims of the FIGO intervention FIGO intervention to increase the capacity of health care professionals to offer PPIUDs by training community midwives, health workers, doctors and delivery unit staff, as appropriate. In other words, the purpose of the 15 month follow up was to see how well the providers were maintaining their capacity to officer PPIUDs. In this instance, a mixed methods study would be very helpful to understand not only what provider performance outcomes are but also why they have changed.

Response: We agree with the reviewer. However, we were able only to replicate the quantitative part of the study among women delivering in the study hospitals in July 2018. We did not interview the providers nor had qualitative interviews with women or providers that would have enriched the study. We have added this limitation in the manuscript (lines 466-469).

**Methods**

Line 141: specially leaflets??? Is this a typo?

Response: Removed the word “specially”.

Line 148-152: What other "contraceptives" are we talking about? This is focused only on IUDs, correct? This paragraph is repetitive and needs to be edited to remove duplication.

Response: Thank you for your suggestions. The text has been revised to clarify and avoid duplication (lines 136-142)

Line 169-174: This paragraph can be simplified as follows:

After the 18-month 170 enrollment period, training of providers, monitoring and technical 173 supervision were stopped and the number of counsellors was reduced from two to one per 174 hospital.

Response: Many thanks for your suggestion. The section has been simplified (lines 155-157).

Line 174-177: delete this section since it is repeated in paragraph immediately after

Response: Thank you for the helpful suggestion. This text has now been deleted.

**Results**

Many of the results related to baseline and intervention have already been reported. See Reprod Health. 2019;16(1), 69 (main study paper); it seems some of the figures in this manuscript are duplicative of what is already published; see specifically Figure 3 PPIUD counseling and Figure 5, PPIUD uptake. Please explain how the figures in the two papers are different.

It seems this analysis is most useful by focusing on the 15 month followup survey to the main intervention. I suggest revising the manuscript to reflect that point and remove all redundancies that are already reported in the main study paper.

Response: Figures 2 and 3 have the added data from the follow up in July 2018 included. This shows a visual of any change since the original study and the follow up. Some text describing these figures has been removed, though some description has been kept in order to make a comparison. We have focussed more on July 2018 follow up.

**Discussion**

Importantly, seeing a drop-off of the intervention once implementation has ended is very common. The more interesting question is why has this happened so framing the discussion more concisely and coherently around this topic would make it easier for the reader to understand.

Response: Thank you for the comment. We have revised the text in Discussion to address this point.

Line 383-384: What is "it"? Seems to refer to different studies; confusing since talking about study results in first part of sentence. Though PPFP knowledge among 383 health workers is shown to increase after the intervention, it has also been shown to reduce as 384 time goes by [28, 29].

Response: Thank you for the comment. ‘It’ here referred to knowledge, as the sentence was discussing the knowledge of providers. We have revised the text (lines 392-395)

Line 400: PPIUD insertion among women in the study hospitals fell significantly after the intervention

401 enrolment. I think just saying the "intervention period" rather than "enrollment" will make it easier for readers to understand.

Response: Thank you, this has now been changed.

**Conclusion**

I agree with reviewer 1 that the issue of comparability of the two groups is important particularly since parity and education are significantly different from the intervention group.

The conclusion that the difference in hospital group uptake related to the length of intervention implementation is purely conjecture since no other attributes to hospital service delivery were assessed.

Response: The baseline characteristics of women in the two groups were balanced at the baseline. Group 1 hospitals received intervention for 9 months compared to Group 2 that received for three months.

**Reviewer #4:** The article was well written and worthy of publication however, there is need for gramatical review and editing.

For example:

line 222- change "in baseline" to "at baseline"

line 230 - 231: the statement "More proportion...to the baseline" seems incomplete.

Response: This statement has been removed.

A thorough read of the manuscript has been completed and edits have been made.
